# Food-related behaviors of rural (Asaase Kooko) and peri-urban (Kaadjanor) households in Ghana

**DOI:** 10.3389/fnut.2025.1523793

**Published:** 2025-02-06

**Authors:** Prince Kwabena Osei, Megan A. McCrory, Matilda Steiner-Asiedu, Edward Sazonov, Mingui Sun, Wenyan Jia, Tom Baranowski, Gary Frost, Benny Lo, Alex Kojo Anderson

**Affiliations:** ^1^Department of Nutritional Sciences, College of Family and Consumer Sciences, University of Georgia, Athens, GA, United States; ^2^Department of Health Sciences, Sargent College of Health & Rehabilitation Sciences, Boston University, Boston, MA, United States; ^3^Department of Nutrition and Food Science, College of Basic and Applied Science, University of Ghana, Accra, Ghana; ^4^Department of Electrical and Computer Engineering, University of Alabama, Tuscaloosa, AL, United States; ^5^Department of Neurological Surgery, University of Pittsburgh, Pittsburgh, PA, United States; ^6^Department of Electrical and Computer Engineering, University of Pittsburgh, Pittsburgh, PA, United States; ^7^USDA/ARS Children’s Nutrition Research Center, Department of Pediatrics, Baylor College of Medicine, Houston, TX, United States; ^8^Section for Nutrition Research, Department of Metabolism, Digestion and Reproduction, Imperial College London, London, United Kingdom; ^9^Hamlyn Centre, Department of Surgery and Cancer, Imperial College London, London, United Kingdom

**Keywords:** food-related behaviors, food acquisition, food preparation, eating practices, food storage, Ghana, rural, peri-urban

## Abstract

**Background:**

“Food-related behaviors” range widely and include food acquisition, storage, cooking, meal sharing, eating practices, among others. Food-related behaviors can influence nutritional status and health outcomes, and likely vary between rural and peri-urban households; however, there is limited documentation on such differences.

**Objective:**

To assess food-related behaviors of rural and peri-urban households in Ghana to inform the design and implementation of a field study to validate passive wearable camera technologies for dietary assessment.

**Methods:**

This was a cross-sectional qualitative study in rural (Asaase Kokoo) and peri-urban (Kaadjanor) communities, Ghana. Forty key informants (20 rural and 20 peri-urban) were interviewed about their household food-related behaviors. All interviews were audio-recorded and transcribed verbatim by professional transcribers, and manually coded using the directed content analysis approach.

**Results:**

All but three key informants were mothers, who were also the main food preparers for their households. The mean (SD) ages of female key informants were 35.5 (19.8) years in rural households and 38.9 (19.2) years in peri-urban households. The ages of two male key informants in rural households were 45 and 60 years, and the age of the only male key informant in a peri-urban household was 53 years. The most prevalent occupation in rural households was farming, while in per-urban households, blue-collar jobs (e.g., beauticians, sales personnel, and commercial drivers) were the main occupations. Farming was the main source of food in rural households, whereas buying food from local markets and grocery stores was the main source of food in peri-urban households. Some in rural and peri-urban households reported that husbands received preferential treatment by being served first with larger meal portions after food preparation in the home. Few key informants in rural households reported that meal-sharing patterns were based on ages of household members, with adults usually served more food than children.

**Conclusion:**

The meal-sharing patterns and eating practices reported in some rural and peri-urban households could potentially impact the nutrition and health of children. Our findings suggest the need for nutrition education for mothers to provide adequate and nutrient-rich foods to support optimal child growth and development.

## Introduction

Food-related behaviors include food acquisition, storage, cooking, meal sharing, and eating practices (1–3). The main factors that influence food-related behaviors are food culture, food environment, knowledge about food, and economic factors (2, 4–7). Food culture includes the traditions and belief systems of populations about food (2, 6, 8). The food environment at a population level is the physical, economic, political, and sociocultural context within which food choices are made (9, 10). A review found that food-related behaviors were predominantly determined by food culture and food environment (6). Understanding the food environment of a population was critical for the effective implementation of nutrition interventions that improved food-related behaviors (9). Individuals’ knowledge about food can influence food-related behaviors (11), and high nutrition knowledge was found to promote healthy eating behavior (12). Similarly, the nutrition knowledge levels of individuals were significantly associated with their healthy food choices and eating practices (7). Environmental factors, such as the availability and accessibility of foods can also impact food-related behaviors (13, 14).

Intra-household meal sharing is usually inequitable, particularly in low-and middle-income countries (LMICs), with children frequently receiving the smallest portions of meals (15–17). Several studies have reported a double burden of malnutrition (coexistence of overnutrition and undernutrition) in the same household, where undernourished children have mothers who are overweight or obese (18–24). Potentially, the double burden of malnutrition could in part be due to food-related behaviors of household members that impede children from receiving adequate food and nutrient intake (25). In Ghana, childhood undernutrition persists in different population sub-groups, with inadequate food intake and poor dietary diversity being the major contributory factors (26–28). High consumption of sugar-sweetened beverages and low consumption of fruit and vegetables have been reported among children (29), and overnutrition thrives among adults in diverse communities (30, 31).

Rural and peri-urban areas in Ghana differ primarily in their proximity to urban areas and their levels of development (32). Rural areas are typically characterized by low population density and large stretches of open land, and the economy is predominantly based on agriculture, forestry, and mining activities with limited access to healthcare facilities, potable water, quality education, and transportation infrastructure (33). Peri-urban areas are transitioning zones between rural and urban areas with increasing urbanization rates (34). They have mixed characteristics of rural and urban areas, and the economy is usually based on diverse business activities with agricultural land giving way to residential, commercial, and industrial developments (34, 35). Also, peri-urban areas have better access to social amenities compared to rural areas (36).

Populations in rural and peri-urban areas tend to encounter different environments that likely influence their food-related behaviors, but little is known in this regard. Since previous studies about food-related behaviors have predominantly focused on urban areas (37, 38), the primary objective of this qualitative study was to examine household food-related behaviors of rural and peri-urban communities in Ghana. Food-related behaviors are shaped by a complex interplay of multiple factors, and using a theoretical framework helps in understanding and analyzing these behaviors more systematically (39). Our guiding theoretical framework for this study was the Cultural Ecological Model, which specifies interactions between culture, environment, and human behavior (36), highlighting the critical role of cultural practices in shaping how individuals interact with their environment. To align with the Cultural Ecological Model, we developed open-ended questions to explore how individual, interpersonal, and community levels of the model interact with culture and the environment to influence food-related behaviors. By examining these interactions, we aimed to gain a deeper understanding of the diversity in food-related behaviors between rural and peri-urban households. We hypothesized there would be differences in food-related behaviors of rural and peri-urban households in Ghana. The findings from this study informed the design and implementation of a field study of passive wearable camera technologies for dietary assessment (40).

## Materials and methods

### Study design and setting

This was a cross-sectional qualitative study. The study duration was from May 2019 to July 2019, which is the major rainy season in southern Ghana. In-depth key informant interviews were conducted with a purposive sample to assess household socio-demographics, food acquisition, preparation, cooking practices, meal sharing, eating practices, and food storage methods of rural and peri-urban households in Ghana. Two different locations were selected, which were rural and peri-urban communities. The choice of rural and peri-urban communities allowed us to capture diversity in culture and socio-demographic characteristics regarding food-related behaviors. For the rural community, Asaase Kokoo in the Akuapem North Municipal Assembly of the Eastern Region of Ghana was selected. Most of the residents in this rural community were engaged in farming. For the peri-urban community, Kaadjanor in the La-Dade Kotopon Municipal Assembly in the Greater Accra Region of Ghana was selected. The majority of residents in this community were involved in blue-collar jobs and trading. The selection of these communities was informed by our research team’s prior experience and ongoing collaborations with community leaders. The characteristics of the populations from these communities were intended to be similar to the communities we aimed to use in our field study to validate passive wearable camera devices for dietary assessment in Ghana.

### Ethical approval

The Human Subjects Institutional Review Board of the University of Georgia (STUDY00006121) and the Institutional Review Board Committee of the Noguchi Memorial Institute for Medical Research at the University of Ghana (#-046/18–19) reviewed and approved the study protocol. Once the study protocol had been explained to interested key informants, and their questions answered, they consented to be interviewed and audio-recorded by either signing or thumb-printing a consent form.

### Eligibility criteria, recruitment, and sample size

To be eligible, a household needed to have at least three members consisting of a mother, father, and an index child (adolescent or child under 5 years), residing in the same household and consuming most of their meals from home. Additionally, a key informant needed to be at least 18 years old and the primary person responsible for household food-related activities, such as food shopping and food preparation. The key informants (usually the mothers) were chosen because it was assumed they would have the best understanding of the food-related behaviors of their households. These eligibility criteria were established to best understand the dynamics around eating practices in rural and peri-urban households. Key informants were invited to participate in the study through word-of-mouth referrals and house-to-house visits, with the assistance of community leaders in identifying eligible households in the rural and peri-urban communities. Purposive sampling was used to select 40 households (20 from each community).

### Data collection

In-depth key informant interviews were conducted using a semi-structured interview script (see Supplementary material) with open-ended questions about household food-related behaviors. The interview script was developed by the research team and pilot-tested in five rural and five peri-urban households in Ghana before the main study. Multi-lingual field staff (i.e., they could speak English and at least one local language) were recruited and trained to administer the interviews. They were also trained on standardized interviewing techniques to limit biases regarding how questions were asked. The field staff worked in pairs during the data collection and where interviews had to be conducted in a local language, they spot-translated the questions on the interview script verbally from English to the local language instantly before asking the participant. Before interviews, key informants completed a brief socio-demographic survey of their households. The field staff then interviewed the key informants by following the interview script focused on the culture around the household’s food acquisition, choice of ingredients, food preparation, meal-sharing patterns, eating practices, food storage, and the overall food environment of the household. The interviews were either conducted in English or a local language (Twi, Ga, or Ga-Adangbe). The brief socio-demographic survey and in-depth interview combined lasted an average of 60 min (range: 45–85 min). Finally, the field staff thanked every key informant with a monetary incentive in the local currency, equivalent to US $20 at the time of the study.

### Data coding and analysis

All the interviews were audio-recorded. They were later transcribed verbatim by professional transcribers and cross-checked with the original recordings (by AKA) to ensure the transcriptions were accurate, complete, and unbiased. Of the 40 key informants who participated in the main study, the audio-recorded files from seven peri-urban households were corrupted therefore, we could not transcribe those files. As a result, transcripts from 33 key informants (20 in rural households and 13 in peri-urban households) were used in data analysis. Transcriptions in local languages were translated into English by professional translators. AKA and PKO read the transcripts multiple times to become thoroughly acquainted with the contents of the transcripts. Then, the transcripts were manually screened and coded independently by PKO to identify the main themes and subthemes using the directed content analysis approach (41). The themes and subthemes developed were cross-checked by AKA and were discussed between PKO and AKA at regular meetings until a consensus was reached. The agreed-upon themes, subthemes, and main findings were summarized (Table 1), and representative quotes were reported.

**Table 1:** Themes, subthemes, and summary of main findings in rural and peri-urban households.

Themes	Subthemes	Rural key informants (*N* = 20)		Peri-Urban key informants (*N* = 13)	
		Percentages and respondents (*n*)	Main findings	Percentages and respondents (*n*)	Main findings
Sources of Livelihood varied by location	Main occupations in rural and peri-urban households	100.0% (20)	Farming (crop farming and livestock rearing). Some household members had other sources of income.	100.0% (13)	Blue-collar jobs (sales personnel, commercial drivers, beauticians, etc.). Some household members had other sources of income.
Household Food Acquisition Practices varied by location	Farming in rural households	100.0% (20)	Crop farming and livestock rearing were the main sources of food.	none	No household acquired food through farming activities.
	Food shopping and frequencies	80.0% (16)	Few food items were shopped from local markets and most households shopped twice per week.	100.0% (13)	All food items were purchased from local markets and grocery stores, with most households shopping between twice a week and daily.
	Food shopping centers	100.0% (20)	Local markets	100.0% (13)	Local markets and grocery stores
	Food shopping items	100.0% (20)	Vegetables (e.g., tomatoes, pepper, onions, etc.), fish, food spices, among others.	100.0% (13)	Staple foods (cassava, maize, plantain, yam), fruit (e.g., orange, pawpaw, etc.), vegetables (e.g., tomatoes, pepper, onions, etc.), processed foods (e.g., sugar-sweetened beverages), food spices, fish, chicken, and meat, among others.
Household Food Preparation Practices varied by location	Household food preparer	85.0% (17)	Mothers were usually in charge of food preparation.	69.2% (9)	Mothers were usually in charge of food preparation.
	Food preparation area	100.0% (20)	Under a shed, kitchen, open space, and on a veranda	100.0% (13)	Kitchen, open space, and on a veranda
	Cooking frequencies	70.0% (14)	Majority cooked twice per day, every day a week	53.8% (7)	Majority cooked once per day, three or four times per week
Household Meal-Sharing Practices varied by members and location	Meal-sharing patterns in rural and peri-urban households	35.0% (7)	Husbands were served first with larger meal portions, meals were shared based on the ages of household members, and adults were usually served larger meal portions.	30.8% (4)	Husbands were served first with larger meal portions and adults were usually served larger meal portions.
Household Eating Practices varied by members and location	Individual-plate eating	20.0% (4)	Husbands, wives, and children ate their individually served meal portions at the same or different times.	23.1% (3)	Husbands, wives, and children ate their individually served meal portions at the same or different times.
	Shared-plate eating	75.0% (15)	Either children and mothers or husbands and wives ate together from the same plate.	61.5% (8)	Either children and mothers or husbands and wives ate together from the same plate.
Methods of Food Storage varied by location	Modern and traditional methods of food storage	15.0% (3)	Very few had refrigerators, and none had a freezer. Some vegetables were boiled to preserve them, meat and fish were preserved and stored by drying and smoking.	100.0% (13)	All households had either a refrigerator or freezer for food storage.

### Statistical analysis

The analysis of socio-demographic characteristics data was conducted using IBM SPSS Software 29.0 (Armonk, NY). Descriptive statistics were used to summarize the socio-demographic characteristics of key informants from rural and peri-urban households, and the results are presented as frequencies, means, and ranges (Table 2).

**Table 2:** Household socio-demographic characteristics of key informants.

Socio-demographic characteristic variable	Rural households (*N* = 20)	Peri-Urban households (*N* = 13)
Number of female key informants	18	12
Number of male key informants	2	1
Age range of female key informants	24–64 years	27–61 years
Mean (SD) age of female key informants	35.5 (19.8) years	38.9 (19.2) years
Ages of male key informants	45, 60 years	53 years
Age range of children <5 years	2 months–4 years	6 weeks–3 years
Range of total number of household members	4–13	4–12
Mean (SD) total number of household members	7.6 (4.8)	7.4 (4.5)
Range of number of children <5 years in households	1–3	1–2
Mean (SD) number of children <5 years in households	2.0 (0.04)	1.0 (0.02)

## Results

### Household socio-demographic characteristics of key informants

The majority of key informants were females in rural and peri-urban households. There were wide ranges in the ages of the female key informants and the mean number of children under 5 years (ages between 2 months and 4 years) in rural households was 2 and the mean was 1 child (ages between 6 weeks and 3 years) in peri-urban households. Details of household socio-demographic characteristics of the key informants are summarized in Table 2.

### Themes, subthemes, and representative quotes

The themes and subthemes that emerged from the interview transcripts are explained below and supported by representative quotes from the key informants in rural and peri-urban households. The percentages and frequencies of key informants, who reported specific food-related behaviors under subthemes are also presented accordingly.

#### Theme 1: sources of livelihood varied by location

Key informants reported diverse sources of livelihood in rural and peri-urban households. Three subthemes emerged under this theme, which are the main occupation in rural households, main occupations in peri-urban households, and other sources of livelihood.

##### Subtheme 1: main occupation in rural households

All key informants reported that farming (crop farming and rearing livestock) was their main occupation. Crops that were mainly cultivated included local staple foods (maize, cassava, plantain, etc.), and livestock reared were mostly pigs, fowl, sheep, and goats.

A quote from a key informant when asked about her occupation was, *“Please he* (husband of key informant) *is a farmer, and I am also a farmer. We grow crops like maize, cassava, and plantain. As for animals, we rear goats and sheep” (rural, 33 years, female).*

##### Subtheme 2: main occupations in peri-urban households

Key informants reported different occupations (differed for males and females), which were mainly blue-collar jobs. Some blue-collar jobs reported were commercial driver, lab technician for males, and females reported food vendors, sales personnel, and beauticians, among others.

When asked about her occupation, a key informant responded, *“Please I sell bottles and sachets of pure water* (treated drinking water)*. I also sell bedsheets and curtains. Please,* he (husband of key informant) *works at [a maternity home]”* (peri-urban, 43 years, female).

##### Subtheme 3: other sources of livelihood

In rural households, some key informants (55.0%, *n* = 11) reported that a number of household members had other jobs apart from their main occupation (farming), which provided additional sources of income for their households.

The response from a key informant was, *“My main occupation is farming. I rear goats and pigs. She* (wife of key informant) *is a trader in provisions. Again, she is engaged in selling food to the school children. She prepares sobolo* (hibiscus drink) *for sale (rural, 60 years, male).”*

Similarly, in peri-urban households, some key informants (46.2%, *n* = 6) reported that some household members had other sources of livelihood apart from their main occupations.

A key informant reported, *“He* (husband of key informant) *works with a lab organization. I have forgotten the name of the organization. It is a private hospital. I am a food vendor. I am a waakye* (local rice and beans meal) *seller”* (peri-urban, 43 years, female).

#### Theme 2: household food acquisition practices varied by location

The sources of food for rural and peri-urban households and the mode of acquisition differed. Five subthemes emerged under this theme: farming in rural households, food shopping and frequencies, food shopping centers, food shopping items, and meal purchases and consumption outside of the home.

##### Subtheme 1: farming in rural households

All rural key informants reported that apart from a few food items that were sometimes purchased from local markets, farming (crop farming and livestock rearing) was their main source of food, and cassava, plantain, maize, and yam were the main staple foods cultivated. Goats, sheep, pigs, and fowl were also the most common livestock reared. Some (40.0%, *n* = 8) reported they usually sold part of their farm produce and kept some for their household consumption.

When asked about the sources of food for a household, a key informant responded, *“We get cassava and plantain from the farm. We sell some and consume part”* (rural, 28 years, female).

Another key informant stated, *“We rear the sheep for sale, but as for the goats we kill some for home consumption. Sometimes when we have visitors, we kill some of the goats, and when we do not have money to buy fish, we kill some of the fowl”* (rural, 37 years, female).

A number of key informants (45.0%, *n* = 9) also reported they cultivated oranges, mangoes, pineapples, and pawpaw, which were usually consumed in season. When asked about fruit consumption in the home, a key informant reported, *“I have pawpaw in my farm. I get oranges during the season. Now it is off-season. The trees are now fruiting. Mango is also off-season. We have all these during the season”* (rural, 51 years, female).

##### Subtheme 2: food shopping and frequencies

Several key informants in rural households (90.0%, *n* = 18) and peri-urban households (77.0%, *n* = 10) reported that mothers were responsible for food shopping. All key informants in peri-urban households reported that shopping for food items from local markets and grocery stores was the main source of food for their household consumption.

A quote from a key informant was, *“We have our food from [local market]. We buy things at [another local market]”* (peri-urban, 61 years, female).

In rural households, a few food items were shopped from local markets. On the question of food shopping, a key informant reported, *“Apart from fish and onions, we do not buy plantain, cassava, or cocoyam from the market; we cultivate them on our farm”* (rural, 34 years, female).

Concerning the frequency of food shopping, most key informants in rural households (80.0%, *n* = 16) shopped twice per week and the remaining shopped less than twice a week. When asked about the frequency of shopping, a key informant reported, *“Please, we do that on Tuesdays and Fridays. These are the market days. Yes, on the market days things sold are cheap”* (rural, 53 years, female).

In peri-urban households, the majority (69.2%, *n* = 9) had a wider range, shopping from twice per week to every day. The remaining shopped in bulk, either once a week or once every 2 weeks. A key informant reported, *“I usually do shopping three times a week. I usually go on Friday, Sunday, and Tuesday. Because I am taking care of the children, I have fixed these days for my shopping”* (peri-urban, 29 years, female).

A quote from another key informant was*, “Please, it is about five times within a week, from Monday to Friday. We can go to the market on weekends sometimes too”* (peri-urban, 45 years, female).

##### Subtheme 3: food shopping centers

In rural households, all key informants reported that sometimes, food items, such as tomatoes, pepper, onions, garden eggs (known as eggplant in the US, and aubergine in the UK and parts of Europe), spices, fish, and salt, among others, were purchased from local markets (i.e., trading centers or marketplaces where a variety of food items, which are mostly fresh local farm produce, and other household items are sold) while in peri-urban households, all key informants reported that they purchased all their household food items from local markets and grocery stores (i.e., retail outlets where a wide range of products, such as frozen meat, dairy, canned and packaged goods, personal care items, and other household items are sold).

A quote from a key informant was, *“He* (husband of key informant) *brings maize, cassava, and so on from the farm. And we buy fish, salt, and other ingredients from the local market”* (rural, 32 years, female).

Another key informant reported, *“I buy meat, chicken, and other things at [grocery store]. It is only on Tuesdays that the foodstuffs from the rural areas are brought to the market for sale”* (peri-urban, 28 years, female).

##### Subtheme 4: food shopping items

In rural households, vegetables, such as tomatoes, pepper, and garden eggs, which were usually not cultivated on their farms, as well as other food items like salt, fish, meat, and food spices, were the main food items that were purchased from local markets. In peri-urban households, the main food items purchased from local markets and grocery stores were: cassava, maize, plantain, yam, fruit, vegetables, processed foods, food spices, fish, chicken, and meat, among others. While no key informant reported purchases and consumption of processed foods in rural households, some in peri-urban households (30.8%, *n* = 4) stated that they shopped for processed foods, such as sugar-sweetened beverages for their household consumption.

When asked about food shopping items in a rural household, a key informant reported, *“Apart from fish, tomatoes, and onions we do not buy others like plantain, cassava, cocoyam from the market. We buy tomatoes, onions, and salt in particular*” (rural, 60 years, male).

The response to the same question from a peri-urban household was, *“Please I buy tomatoes, pepper, onions and sometimes cassava, plantain and corn dough, milo, and others from [local Market]”* (peri-urban, 46 years, female).

Another key informant reported, *“I buy rice, canned drinks, and biscuits from [grocery store] and corn dough, yam and others from the local market”* (peri-urban, 28 years, female).

##### Subthemes 5: meal purchases and consumption outside of the home

Some key informants in rural households (60.0%, *n* = 12) and all key informants in peri-urban households reported that, apart from their main meals that were usually prepared in the home, some meals were purchased from food vendors outside of the home.

When asked if some meals were purchased outside of the home in a rural household, a key informant reported, *“We buy food in the afternoon. We usually buy fried yam and sometimes we buy gari, sugar, and groundnuts”* (rural, 36 years, female).

Similarly, the response of a key informant in a peri-urban household to the same question was, *“I do not have time to prepare meals in the morning. So, in the morning they buy food and take it to school. Sometimes I go out to buy a canned drink and bread to eat”* (peri-urban, 36 years, female).

In addition, some in rural households (45.0%, *n* = 9) and peri-urban households (38.5%, *n* = 5) reported that their children, who were enrolled in public schools, were fed meals by their schools during lunchtime.

A quote from a key informant was, *“The school feeds them in the afternoon. Please, one of my children does not eat the food provided by the school. Sometimes she sends food from home to school or sometimes buys something there”* (rural, 42 years, female).

A quote from another key informant was, *“The children eat at school. They give them lunch. That’s the school feeding program”* (peri-urban, 42 years, female).

#### Theme 3: household food preparation practices varied by location

Key informants reported some similarities and differences in food preparation practices in rural and peri-urban households. Three subthemes emerged: household food preparer, food preparation area, and cooking frequencies.

##### Subtheme 1: household food preparer

In most rural households (85.0%, *n* = 17) and peri-urban households (69.2%, *n* = 9), mothers were usually in charge of food preparation.

A key informant reported, *“It is the woman who cooks. If the woman is not available, sometimes when I come from the farm, I put the food on fire and later, she comes and finishes the cooking”* (rural, 60 years, male).

Another quote from a key informant was, *“Most times, it is me, except on Sundays. I am a makeup artist, so I am very busy on Saturdays and Sundays. When I leave home for an appointment, I ask my sister to cook, but from Monday to Friday I do all the cooking”* (peri-urban, 42 years, female).

##### Subtheme 2: food preparation area

Food preparation areas in rural households were: under a shed (15.0%, *n* = 3), in a kitchen (40.0%, *n* = 8), in an open space (35.0%, *n* = 7), and either in a kitchen or outside on a veranda (10%, *n* = 2). In the peri-urban households, food preparation areas were: in a kitchen (38.5%, *n* = 5), in an open space (46.2%, *n* = 6), and either in a kitchen or outside on a veranda (15.4%, *n* = 2).

A quote from a key informant in a rural household was, *“Please, we cook outside there. We have made the hearth outside”* (rural, 26 years, female).

*“I cook in the kitchen. It is in the room. I can also cook outside. When I want to prepare banku, I do it outside because I cannot use the cooker”* (peri-urban, 52 years, female), another quote in a peri-urban household.

##### Subtheme 3: cooking frequencies

Cooking frequencies varied in rural and peri-urban households. Frequencies reported were higher in rural households than in peri-urban households. The majority of key informants in rural households (70.0%, *n* = 14) cooked twice, every day a week. The remaining cooked three times, every day a week. In peri-urban households, most key informants (53.8%, *n* = 7) cooked once per day, three or four times per week, and the remaining reported lower cooking frequencies per week.

A key informant in a rural household reported, *“As for cooking, I will say we cook every day, but mostly two times, in the morning and evening”* (rural, 57 years, female).

A quote from a key informant in a peri-urban household was, *“Nhmm, I cook once per day four times a week. It is not always that I prepare breakfast and lunch”* (peri-urban, 27 years, female).

#### Theme 4: household meal-sharing practices varied by members and location

Key informants reported different patterns in how meals were shared after food preparation among husbands, mothers, and children in rural and peri-urban households. Two subthemes emerged: meal-sharing patterns in rural households and meal-sharing patterns in peri-urban households.

##### Subtheme 1: meal-sharing patterns in rural households

Many key informants (85.0%, *n* = 17) reported meals were usually served by mothers after food preparation in the home. Some key informants (35.0%, *n* = 7) reported that their husbands were served first with larger meal portions. This practice was mainly because husbands were considered the heads of their households, the primary breadwinners, and provided the money for food shopping, therefore, they deserved the largest portions of meals all the time.

A quote from a key informant was, *“Hmm. Honestly, when I cook the food, I give him* (husband of key informant) *more because he is the head, so the children cannot eat what he eats. So, I give him more than I give to the children”* (rural, 48 years, female).

A few key informants (20.0%, *n* = 4) also mentioned that meals were shared based on the ages of household members, and adults were usually served larger meal portions than children: the primary reason being that adults must be respected for their seniority.

A key informant reported, *“Please, the younger children are given their share according to their ages and the adults too. The older ones eat theirs together”* (rural, 36 years, female).

##### Subtheme 2: meal-sharing patterns in peri-urban households

Some key informants (46.2%, *n* = 6) reported meals were usually served by mothers after food preparation. A few key informants (30.8%, *n* = 4) stated their husbands were served first with larger meal portions.

A representative quote from a key informant was, *“Please, I serve my husband first. After him I serve my firstborn, then the secondborn, the thirdborn, followed by the twins before I serve myself. If we prepare fufu* (typical West African dish made from cassava, plantains, or cocoyam, boiled and pounded into a smooth dough-like consistency, and eaten with light soup, palm nut soup, or groundnut/peanut soup and any protein of choice)*, I give my husband a special quantity of fish, and the children are also given little of it”* (peri-urban, 42 years, female).

#### Theme 5: household eating practices varied by members and location

There were similar patterns in how eating practices occurred among household members (husbands, wives, and children) in rural and peri-urban households. While some household members shared their meals, others did not and consumed their meals separately. Two subthemes emerged: individual-plate eating and shared-plate eating.

##### Subtheme 1: individual-plate eating

A few key informants in rural households (20.0%, *n* = 4) and peri-urban households (23.1%, *n* = 3) reported that after meal preparation in the home, husbands, wives, and children ate their individually served meal portions at the same or different times.

A quote from a key informant was, *“I eat alone. That is how I have been brought up”* (rural, 52 years, female).

A key informant reported, *“I call everybody to pick their share. Please, everyone sits at a table and eat their shares. We have plastic tables. My husband may not be ready by the time I finish cooking, so I serve him separately”* (peri-urban, 27 years, female).

##### Subtheme 2: shared-plate eating

Several key informants in rural households (75.0%, *n* = 15) and peri-urban households (61.5%, *n* = 8) reported that either children and mothers or husbands and wives ate together from the same plate.

A quote from a key informant was, *“We all eat together whereby I do not dish it out to them individually. If somebody is not around, we reserve that person’s share for him or her. I and the other children eat together”* (rural, 26 years, female).

Another key informant reported, “*When we have banku* (typical Ghanaian dish made from a mixture of fermented corn and cassava dough, cooked into a smooth elastic consistency and eaten with soups, stews or pepper sauce and any protein of choice) *or rice, they pick a big bowl like the one you see. Sometimes I join the children and we eat together”* (peri-urban, 45 years, female).

#### Theme 6: methods of food storage varied by location

Key informants in rural and peri-urban households reported different ways of storing food. These methods included modern and traditional techniques for storing mainly staple foods, vegetables, fish, and meat. Two subthemes emerged: modern methods of food storage and traditional methods of food storage in rural households.

##### Subtheme 1: modern methods of food storage

In peri-urban households, all key informants reported they had either a refrigerator or freezer for food storage, but in rural households, only 15.0% (*n* = 3) had refrigerators for food storage and none reported having a freezer.

A quote from a key informant in a rural household was, *“Since I do not have a fridge, I buy tomatoes that I can consume within 3 or 4 days. As for the chicken, I have some fowl at home so I can kill any of them as and when I like*” (rural, 39 years, female).

One key informant in a rural household reported sometimes she kept vegetables in the refrigerator of a friend (rural, 26 years, female).

A key informant in a peri-urban household reported, *“Storage? I have a deep freezer. Yes, I have a fridge. When I buy meat and fish, I keep them in the freezer. We keep those that will decay in the refrigerator”* (peri-urban, 61 years, female).

In peri-urban households, all key informants reported vegetables, meat, and fish were stored in refrigerators or freezers to preserve them.

*“The yam I put them on a wood under the table and then the kontomire* (green leafy vegetable) *are the ones I put them in the fridge”* (peri-urban, 50 years, female).

Another quote was, *“For the meat and fish, I use what I need and keep the rest in a refrigerator, since they could easily go bad, So I pick them from the fridge when I need them”* (peri-urban, 43 years, female).

##### Subthemes 2: traditional methods of food storage in rural households

All key informants reported that local staple foods, such as cassava, plantain, yam, and maize were stored either in sacks or containers in a kitchen or open space. A few key informants (15.0%, *n* = 3) also stated that they stored cassava in water to preserve it.

The response of a key informant was, *“We put them in a sack. The cassava, at times we put them in water to prevent decay. We keep them in the kitchen. They would decay if you put them on the bare cemented floor”* (rural, 28 years, female).

The majority of key informants (85.0%, *n* = 17), who did not have refrigerators, reported that they stored vegetables like onions, peppers, and tomatoes in baskets and polyethylene bags or boiled the vegetables to preserve them.

To the question of how vegetables and fruits were stored, a key informant reported, *“As for tomatoes, we grind them and boil the paste to preserve it. As for the fruits, we eat them the very day they are harvested. No, we do not have a refrigerator”* (rural, 26 years, female).

Some key informants (55.0%, *n* = 11) reported they stored fish and meat by smoking and drying them. *“When we buy fish and meat in large quantities, we smoke those that are likely to decay so that it does not decay”* (rural, 24 years, female).

## Discussion

We examined the food-related behaviors of selected rural and peri-urban households in two communities in Ghana. We found similarities and differences in the sources of food for household consumption, meal-sharing patterns and eating practices. While rural households mainly acquired food from their own household farming activities, peri-urban households relied entirely on purchasing food from local markets and grocery stores. We found adults were usually served larger meal portions compared to children, after food preparation in rural and urban households. The practice of sharing meals with other household members were noted in both rural and urban households. These findings informed the selection of the appropriate wearable camera technologies that were used to capture food consumption activities of each household member (father, mother, adolescent, and child under 5 years) in our field study to validate these innovative technologies for dietary assessment. The meal-sharing patterns and eating practices documented from this study could inform future studies regarding the best strategies to use to measure food consumption activities more accurately in diverse populations.

Farming was the main occupation in rural households aligns with a study that also identified farming as the primary occupation in rural communities in Ghana and other developing countries in Sub-Saharan Africa (42). However, our study adds to literature by highlighting additional sources of income for rural households in Ghana apart from farming, which were usually selling of cooked meals to the general public. The majority of occupations in peri-urban households were blue-collar jobs, because there are more blue-collar job opportunities in peri-urban areas (43). A study found individuals living in peri-urban areas were more likely to be employed in blue-collar jobs (44, 45). In Ghana, there has been a significant upsurge in the rate of migration from rural areas to peri-urban and urban areas in search of jobs and other economic opportunities (46). A review found the migration of rural residents to peri-urban and urban areas posed nutrition and health threats to many populations because dietary patterns changed from traditional diets (mainly of complex carbohydrates, whole cereals/grains, fruit, and vegetables, and low animal-based foods) to consumption of energy-dense processed foods and low intake of fruit and vegetables (47). A higher prevalence of overweight and obesity have been reported in peri-urban populations compared to rural populations (48). As a result, peri-urban residents may be at higher risk of nutrition-related diseases associated with overweight and obesity. To address this, our findings provide insights into the dynamics around the sources of food in peri-urban areas for the Government of Ghana to strategize and prioritize interventions that integrate peri-urban planning, nutrition, and health, while creating job opportunities to motivate populations to stay in rural areas.

Most mothers in rural and peri-urban households were in charge of food shopping, consistent with a study that reported that women were responsible for food shopping-related activities, such as identifying where to purchase food, arranging shopping trips, and deciding what types of meals to prepare in the home to feed their household members (49). A review found that Ghanaian women had a low understanding of food and nutrition labels when making food purchases for their households (50). Mothers in our study had a significant influence on the food choices of their households. Therefore, educating them about healthier food choices regarding the selection of foods that will provide their families essential nutrients while reducing excessive intake of added sugars, saturated fats, and excessive calories, could improve the overall nutrition and health of their families. We found peri-urban households were exposed to processed foods, as some key informants reported consumption of sugar-sweetened beverages and biscuits (cookies). Peri-urban households also shopped for food more frequently than rural households. These findings could be because peri-urban households acquired foods entirely by purchasing from local markets and grocery stores (more processed foods are usually sold in grocery stores), unlike in rural households where the majority of foods consumed were acquired or gathered from the farms of households. In Ghana, despite the Government’s efforts to promote healthy food environments in urban and peri-urban areas, a Healthy Food-Environment Policy Index assessment revealed shortcomings in the implementation of recommended policies (51). Therefore, stakeholders in nutrition and health must establish robust strategies and actions that would ensure the successful implementation of policies to promote healthy food environments in urban and peri-urban areas of Ghana. Our findings show that making local markets, where fresh farm produce are sold more accessible, while limiting the sale of processed foods could improve the food environment in peri-urban areas.

Finding that the majority of mothers in rural and peri-urban households were in charge of food preparation in their households was not surprising because culturally, cooking household meals has been earmarked as the responsibility of a woman, particularly in LMICs (49). However, men have recently been involved in the preparation of meals at home, although women still cook household meals more than men (52). The involvement of men in meal preparation at home could be partly due to the involvement of women in the workforce. In the past, women were denied formal education and therefore, the opportunity to contribute to household income and the development of their countries (49). A study found women with less education were more likely to spend more time cooking household meals than women who had higher education (52). Concerning food preparation in the home, rural households prepared their meals either under a shed, in a kitchen, or in an open space, while peri-urban households prepared meals either in a kitchen or an open space. The frequency of home cooking reported in rural households was higher compared to peri-urban households. A study observed households that cooked more often at home were more likely to make healthier food choices (53). Finding that rural households cooked more frequently than peri-urban households could be because household members in peri-urban communities were employed in blue-collar jobs hence, they had busy work schedules, spent more time away from home, and therefore, did not have much time to cook compared to their rural counterparts whose farming activities were not far from their homes.

In about a third of rural households and a third of peri-urban households, husbands were served larger meal portions than other household members because they were considered the heads of their households and provided the money for food shopping and therefore, deserved the largest portions of meals all the time. Some key informants in rural households also reported that meal-sharing patterns were based on the ages of household members, and adults were typically served more food than children because they were regarded as seniors deserving of respect. Shared-plate eating is a common practice in Ghana and other LMICs where two or more people eat main meals (breakfast, lunch, or dinner) directly from the same plate or bowl (54, 55). Our study documented the practice of shared-plate eating, which was more frequently reported by rural households than peri-urban households. Individual-plate eating was reserved primarily for husbands and adults, who were usually not at home by mealtime, especially in peri-urban households, except mothers who often shared their meals with young children. Our findings contribute to the literature on meal-sharing and eating practices rural and peri-urban households in Ghana. However, the meal-sharing patterns and eating practices are common in many LMICs and have the potential to contribute to the double burden of malnutrition at the household level: children may become undernourished because they are not fed adequately due to inequities in meal sharing among household members that disadvantaged them, compared with other household members who do not share their meals, consume more food, and are overweight or obese (15, 24, 56, 57). A study found about 27% of households had at least one household member who was overweight, and a child, who was either stunted, wasted, or underweight living in the same household (58), and the prevalence of the double burden of malnutrition at the household level in another study ranged from 13 to 17% in some rural communities (59). To address the inequities in meal sharing and eating practices, nutrition programs to educate mothers in rural and peri-urban communities will help them to understand the importance of feeding their children with adequate nutrient-rich foods to support optimal growth and development.

While most rural households relied on traditional methods for preserving their foods, all key informants in peri-urban households had either a refrigerator or freezer for food storage. Another study found refrigerators and freezers were the mundane household food storage appliances for food storage in many urban and peri-urban populations (60). Refrigerators were also found to be the first assets many households bought in urban and peri-urban communities before purchasing other household assets (61). In a little over half of rural households, most perishable foods, such as meat and fish were preserved by smoking, and staple foods like maize were also stored and preserved by drying. This is consistent with drying being the main method for storing cereals and legumes in rural areas (62). In other studies, smoking was the most common technique for preserving and storing fish and meat in rural communities (63, 64).

The findings from this study informed the design and implementation of a field study to validate passive wearable technologies (Automatic Ingestion Monitor version-2, e-Button, e-Hat, and FoodCam) for dietary assessment in Ghana (40). The household food-related behaviors identified guided the selection of the appropriate camera technologies to capture the dietary intake of each household member (father, mother, adolescent, and child under 5 years). The meal-sharing patterns and eating practices in households informed the strategies that were adopted to use other traditional dietary assessment methods (weighed food records and image-assisted 24-h recall) to measure food consumption activities in households.

### Strengths and limitations

A major strength of this study was that we interviewed the main primary food preparers, who had in-depth information about their household food-related behaviors. Another strength was the inclusion of households with at least one index child (child under age 5 years or adolescent), which provided deeper insights into the dynamics around household characteristics. Also, there is dearth of information about food-related behaviors of rural and peri-urban households in Ghana, therefore, we included two different geographical locations in the study, and this helped to capture the diversity in food-related behaviors in rural and peri-urban communities. However, we acknowledge that the study had some limitations. The sample size was small. Also, seven audio-recorded files from peri-urban households were corrupted and could not be transcribed, and the study conducted in only one rural and one peri-urban communities in southern Ghana. Thus, our findings may not fully represent the food-related behaviors of both rural and peri-urban households in Ghana. Moreover, we could not assess the impact of seasonal variations on our findings, which could potentially vary in the two communities. These limitations may reduce the generalizability of the findings to rural and peri-urban communities in Ghana. Nevertheless, our findings contribute to the literature and provide insights into the diversity in food-related behaviors of rural and peri-urban households in Ghana, which are relevant for planning strategies for the design and implementation of nutrition intervention programs in these areas.

## Conclusion

We found similarities and differences in food-related behaviors of rural and urban households. In some rural and peri-urban areas, the meal-sharing patterns and eating practices reported may negatively impact children’s nutrition and health, as children often receive smaller meal portions after food preparation. Nutrition education programs are needed to help mothers to understand the importance of providing their children with adequate nutrient-rich foods to support optimal growth and development. The findings guided the choice of wearable passive camera technologies that were used a field validation study of food consumption activities in Ghana. The documented food-related behaviors provide valuable context for future studies aiming to use innovative technologies for dietary assessment among free-living individuals in rural and peri-urban settings. Also, our findings could guide strategies for implementing nutrition interventions, particularly in rural and peri-urban areas of Ghana. Additional studies are needed to further examine food-related behaviors in other rural and peri-urban areas of Ghana.

## Data Availability

The raw data supporting the conclusions of this article will be made available by the authors, without undue reservation.
