# Supplementary material for: Food-related behaviors of rural (Asaase Kooko) and peri-urban (Kaadjanor) households in Ghana
Source: Front Nutr. 2025 Feb 6;12:1523793. doi: 10.3389/fnut.2025.1523793 (PMC11839426; doi:10.3389/fnut.2025.1523793)
Supplement: Supplementary file 1 [file Table_1.docx]

**Questions for In-depth Key Informant Interview**

1. How many people are in your household? Please provide a description for each member of the household in terms of age, gender, and relationship, etc.
2. What is your experience with dietary assessment?

*Probe:*

1. Where does your household generally obtain the majority of your food?

*Probe:*

1. Do you want to describe how the food is acquired and the frequency at which this is done?

*Probe:*

1. Do you have storage facilities within your household where you keep food?

*Probe:*

1. Who generally prepares meals in your household and how often is food prepared per week?

*Probe:*

1. Where does food preparation occur in your household? Is it an open or closed place?

*Probe:*

1. Who shares meals in your household and where does this take place?

*Probe:*

1. Do you want to describe how and where meals are eaten in your household?

*Probe:*

1. Can you tell us about the number of times you eat in a day and the times each normally occurs?

*Probe:*

1. Do you want to describe your typical day from the time of wake up through the time you go to bed?

*Probe:*
